# Analyses of Nogo-Family Genes in Mouse and Human Microglia Omics Datasets Identify *LINGO1* as a Candidate Drug Target in Alzheimer’s Disease

**DOI:** 10.2174/011570159X359944250722061312

**Published:** 2025-07-30

**Authors:** Elliot J. Glotfelty, Tobias E. Karlsson, Luis B. Tovar-y-Romo, Lars Olson, Brandon K. Harvey, Nigel H. Greig

**Affiliations:** 1 Cellular Stress and Inflammation Unit, Integrative Neuroscience Department, National Institute on Drug Abuse, National Institutes of Health, Baltimore, Maryland 21224, USA;; 2 Department of Neuroscience, Karolinska Institutet, Stockholm, Sweden;; 3 Division of Neuroscience, Institute of Cellular Physiology, Universidad Nacional Autónoma de México, Mexico City, Mexico;; 4 Drug Design & Development Section, Translational Gerontology Branch, Intramural Research Program National Institute on Aging, NIH, Baltimore, MD21224, United States

**Keywords:** *LINGO1*, nogo, microglia, ribotag, CZ cellxgene discover, TACA, transcriptomics, inflammation, Alzheimer’s disease

## Abstract

Microglia are the innate immune cells of the brain. Recent single cell and nucleus sequencing along with other omics technologies are leading the way for new discoveries related to microglial function and diversity. The Nogo-signaling system is a prime target for investigation with these tools as it has previously been neglected in microglia. The Nogo-signaling system consists of approximately 20 proteins, including ligands, receptors, co-receptors, and endogenous inhibitors known for their neuronal plasticity restricting properties *via* RhoA and ROCK1/ROCK2 activation, and have recently been implicated in microglial function. Here, we explore expression patterns of Nogo-family genes in the mouse and human brain. In mice, we focus on brain cell type enrichment, patterns of expression in microglia from embryonic stages to adulthood, sex differences, and changes in expression in acute and chronic inflammatory contexts from publicly available RNAseq and RiboTag translational profiling datasets. We identified differential expression of Nogo-family genes across age, sex, and disease/injury in mice. To analyze human microglia, we utilize a new tool, the *CZ CellxGene Discover*, to aggregate 21 single cell sequencing datasets of human brain cells in Alzheimer’s (AD) and control patients. In humans, *LINGO1* is highly enriched in human AD microglia, a previously undescribed finding. We used *The Alzheimer’s Cell Atlas* (TACA) to further verify if this enrichment correlates to disease state, severity of human AD diagnosis, or sex of patients. The current work provides a comprehensive analysis of Nogo-family genes in microglia and identifies *LINGO1* as a potential therapeutic target for AD.

## 1 INTRODUCTION

Brain function is critically reliant on the precise assembly, maintenance, and plasticity of the intricate synaptic network. During development, an initial scaffold of neural circuits is laid down and then shaped by experience-driven neural plasticity. This decline occurs after an early postnatal critical period, leaving limited but crucial neural network plasticity to optimize microcircuits within the mature brain in response to changing conditions, such as the formation of lasting memories [1-3]. This restricted plasticity stabilizes the neuronal architecture but can limit functional recovery following a CNS insult, such as a stroke or Traumatic Brain Injury (TBI), as well as aggravate neurodegenerative disorders such as Alzheimer’s Disease (AD). A diverse group of molecules is known to limit adult structural synaptic plasticity.

**Fig. (1):**
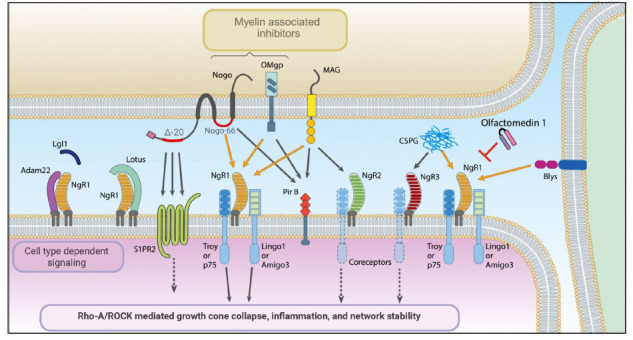
The Nogo signaling system ligands, receptors, co-receptors, and endogenous inhibitors. Nogo signaling broadly results in RhoA activation, initiating a variety of downstream cellular responses. The Nogo-signaling system acts as a stabilizing network of proteins, but can prevent repair or injury resolution, with detrimental effects in chronic brain diseases. Figure adapted from Karlsson *et al*. (2017) with permission. Illustration by Annica Röhl.

Particularly important is the Nogo-like signaling system and the myelin- associated inhibitors (MAIs) including Nogo, myelin-associated glycoprotein (MAG), and oligodendrocyte myelin glycoprotein (Omg) [4]. Nogo originates from the reticulon 4 (RTN4) gene and is expressed as either Nogo-A, -B, or -C, all three of which share a highly conserved C-terminus and a 66-amino acid signaling domain known as Nogo-66. All three isoforms are highly expressed on cell surfaces and in the endoplasmic reticulum [5]. The signaling of Nogo-66, MAG, and Omg is most prominently mediated by the glycosyl-phosphatidylinositol-anchored Nogo receptor 1 (NgR1), with several coreceptors and modulators also participating [6]. The total Nogo-signaling system comprises upwards of 20 different proteins, including ligands, receptors, co-receptors, and endogenous inhibitors (Fig. **1**) [7]. Nogo-A has an additional signaling domain known as Δ-20, or amino-Nogo, that activates sphingosine 1-phosphate receptor 2 (S1PR2) [8]. Despite differences in ligands, receptors, and co-receptors, overall Nogo-signaling converges on a similar intracellular pathway, activating the GTPase ras homolog gene family member A (RhoA) [9] and downstream Rho-associated coiled-coil containing protein kinases 1 and 2 (ROCK1 and ROCK2) (Fig. **1**). Importantly, RhoA initiates diverse and cell type-dependent signaling [10, 11] with many downstream targets [12]. The Nogo-signaling system has been primarily described in relation to its synaptic plasticity restriction properties in neurons; however, there is increasing evidence that this protein family may be involved with microglia, the innate immune cells of the brain responsible for initiating inflammation cascades in the brain.

Neuroinflammation arising from microglia is recognized as a primary cause and symptom of nearly all brain injuries and chronic diseases. Notably, a recent Genome-Wide Association Study (GWAS) found that AD and related dementias may be driven by the elevation of inflammation-related gene expression pathways [13]. Microglia are highly motile cells that comprise approximately 10% of the total cells in the brain. Despite their limited occupation of the brain, microglia also recruit peripheral immune cells that can contribute to neuroinflammation and the containment of Central Nervous System (CNS) damage. Although neuroinflammation is typically described in association with its pathological effects, especially in the context of prolonged injuries or disorders, the process is also crucial for a variety of positive physiologic responses, including neurorepair [14].

The Nogo-signaling pathway has recently been implicated in neuroinflammation [15, 16], exacerbating inflammatory signaling in both primary and immortalized microglia, as evidenced by increased production of two prominent proinflammatory cytokines, Tumor Necrosis Factor-α (TNF-α) and Interleukin-6 (IL-6) [17]. Commercially available Nogo-66 (Nogo-P4) peptide added to immortalized microglia also significantly increased inflammatory protein production when combined with a low concentration lipopolysaccharide challenge. In addition, mice with conditional deletion of microglial Nogo have been produced (MinoKO mice), which show signs of decreased microglial and astrocytic activation and improved recovery following a Controlled Cortical Impact (CCI) injury. This further evidence suggests that microglial Nogo negatively affects morbidity following brain injury [18]. Antibodies and antagonists against Leucine-rich repeat and immunoglobulin-like domain-containing nogo receptor-interacting protein 1 (*LINGO1*), a prominent co-receptor for NgR1, reduce microglial activation and cognitive deficits, respectively, in the APP/ PS1 mouse AD model [19, 20]. Several human AD studies have reported elevated *LINGO1* levels in oligodendrocytes and neurons [21, 22], particularly in females [23]. However, *LINGO1* involvement in microglia has been limited to investigations in Multiple Sclerosis (MS) [24]. Recent studies have highlighted the role of Nogo-A in inducing tauopathy vulnerability [25], while pharmacologically blocking Nogo signaling provides a therapeutic strategy to mitigate amyloid-beta-induced neurite outgrowth inhibition [26].

To examine the Nogo-family of genes in microglia, publicly available OMICS datasets were utilized to investigate the enrichment of Nogo-signaling genes in mouse and human microglia. Other cell types are also included in some analyses. In mice, microglial expression patterns were examined from embryonic stages to adulthood, and changes in translationally relevant genes in response to acute inflammatory challenges, specifically Lipopolysaccharide (LPS) and *polyinosinic-polycytidylic acid* (Poly (I: C)) as well as AD models. In addition, new tools, like the *Chan Zuckerberg (CZ) CellxGene: Discover Platform* [27] and *The Alzheimer’s Cell Atlas* (*TACA*) [28], were utilized to examine human microglia. The *CZ CellxGene: Discover Platform* aggregates single cell/nuclei datasets across many different tissue types and diseases, allowing us to probe broad microglial Nogo-family gene enrichment in human microglia during AD and non-pathological states. *TACA* is a similar platform focused on providing a user interface for analyzing individual single-cell/nucleus datasets related to AD. In mice, several Nogo-family genes, including *RTN4* (Nogo), Nogo receptor-3 (*Rtn4rl1* or *NgR3*), *Mag*, *S1pr2*, syndecan-3 (*Sdc3*), syndecan-4 (*Sdc4*), and *Lilrb3* (PirB), are enriched in mouse microglia or are regulated during an inflammatory insult, with several others differentially regulated based on sex and age. Notably, *RTN4* and especially *LINGO1* emerge as highly expressed Nogo-family genes in human AD microglia.

Differentially Expressed Gene (DEG) analysis of two independent studies [29, 30] available in the *TACA* database shows opposite expression patterns of *LINGO1*. Specifically, Leng *et al*. (2021) [29] highlight the increasing severity of AD, correlating with a significant decrease in *LINGO1* expression in microglia and other cell types. In contrast, Grubman *et al.* (2019) [30] describe the significant upregulation of *LINGO1* in AD patients, as well as sex differences in expression. This study provides the first comprehensive in-depth look at cell type-specific Nogo-family gene expression in mouse and human pathology. The focus on microglia-specific regulation led to an even more detailed analysis of other cell types, illuminating Nogo-family gene *LINGO1* as a possible novel target for drug development across many cell types in AD pathology. With increasing uncertainty about funding expenditures for basic research and the competitive nature of such grants, especially from the US government, the use of free online OMICs platforms will progressively become an essential tool for scientists. These platforms can vastly drive the cost of research down and potentially yield new and relevant findings without expensive equipment or techniques.

## 2 METHODS

### 2.1 Choosing Datasets and Limitations

The current work utilizes publicly available datasets with user interfaces accessible to all researchers or interested parties, particularly those without the software or coding skills typically required to assess these large-scale studies. Using these datasets demonstrates a democratization of science, which includes literature that is often challenging to understand and supplementary file types containing data that have not been fully explored previously. Microglia were of particular interest in the current work, as descriptions of the expression data for the genes of interest (Nogo family genes) in microglia are mainly absent from the literature. All datasets primarily focused on microglia or included microglial cells in the overall study. Importantly, this work uses datasets that incorporate a variety of methods to evaluate gene expression with a focus unrelated to the current research question. All gene expression data were collected in an unbiased manner, and it is acknowledged that not all datasets available were used in this evaluation. Human data of healthy and diseased brains are not widely available, especially within the limited confines of analyzing microglial populations in defined disease states, ages, or sex. To understand the overall big picture of how Nogo-family genes may affect microglial phenotypes in mice, we sought data that provided basal expression across the mouse lifespan in both males and females, as well as in disease and inflammation models. To assess human data, the *CZ Cell×Gene* database was utilized, which aims to collate all single-cell RNA sequencing data published across multiple journals in diverse tissues and disease contexts. This resource is particularly unique in that it allows users to probe specific cell types across a wide variety of conditions. The initial analysis of the *CZ Cell×Gene* collated data was followed by an investigation using a disease-specific database, *The Alzheimer’s Cell Atlas (TACA).* This database enabled us to assess individual datasets that investigated microglia in Alzheimer’s disease at various stages of the disease and across sexes. The analysis was limited to two studies that had clear demarcation of microglia and relevant comparators. The current work should not be considered conclusive, but rather can be used as a resource and model for future studies that probe specific gene sets.

### 2.2 Brain Cell-Type Specific Enrichment of Nogo-Family Genes

The web platform www.brainrnaseq.org was used to access [31, 32] brain cell type enrichment of Nogo-family genes in the mouse cerebral cortex. Sex of animals was not specified in the dataset. The purification protocols for glial cells, astrocytes, and vascular cells are from previously published dissociation, immunopanning, and fluorescence-activated cell sorting protocols [33, 34]. For cell type enrichment analysis, two mice were used from ages P7-P17 [31]. For E17-P60 mice, n = 2 experiments of two animals (P10 and older) or one litter (under P10) for each age/condition, except for P7 (n = 3), P21 (n = 3), and P60 (n = 3) [32]. Total FPKM graph was generated by adding FPKM of all cell types (100% of expression). This “Total FPKM” for each gene was then divided by the contribution of each cell type to visualize cells with high enrichment of Nogo family genes.

### 2.3 RiboTag Translational Profiling

**Fig. (2):**
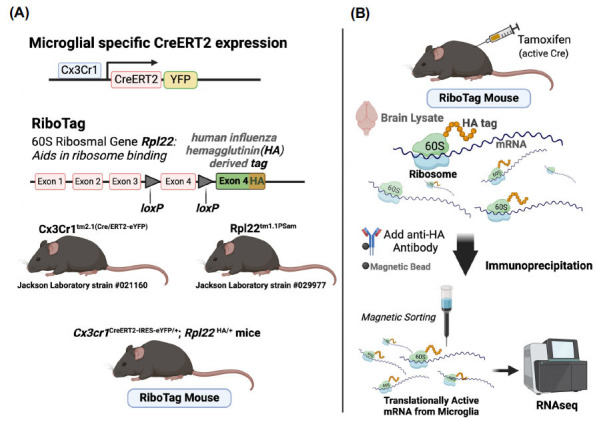
RiboTag transgenic mice were used in the studies by Kang *et al*. (2018). Breeding paradigm to generate microglial-specific HA-tagged RPL22 (**A**), allowing for generation of RNAseq data containing only mRNA that is actively translated at the time of extraction (**B**). Created in BioRender. Glotfelty, E. (2024) BioRender.com/a92b383.

RiboTag [35] data from the Fryer Lab (fryerlab.com/ribotag) were used to assess translationally relevant microglial Nogo-family genes based on sex, aging, and acute (LPS and Poly(I: C)) and chronic (APP/PS1 and AAV-Tau-induced tauopathy) AD models [36]. The acute and chronic models of inflammation utilized only male mice. Microglial RiboTag mice are created by breeding the *Cx3cr1^CreERT2-eYFP/+^* mice (Jackson Labs stock#021160) with the RiboTag mouse line Rpl22^tm1.1PSam^ (Jackson Lab stock #029977). The *Cx3cr1^CreER-YFP+/-^* mouse has tamoxifen inducible Cre recombinase (CreER) and EYFP knocked into the Cx3cr1 locus, a gene specific to microglial cells of the brain and peripheral macrophages (Fig. **2A**). The RiboTag mouse utilizes a modified 60S ribosomal gene, *Rpl22*, so that in the presence of Cre recombinase, Exon 4 of *Rpl22* is replaced with an HA-tagged Exon 4 (Fig. **2B**). The HA-tag is derived from the human influenza Hemagglutinin (HA) protein (amino acids YPYDVPDYA). The HA-tagged Exon 4 labels microglial ribosomes, allowing for immunoprecipitation *via* anti-HA antibodies with a magnetic bead attached. Magnetic Activated Cell Sorting (MACS) was then used to separate mRNA transcripts with HA-tagged ribosomes attached (Fig. **2B**). This method offers several advantages over traditional RNA-seq, including the easier isolation of mRNA transcripts by cell type and the sequencing of mRNA species actively undergoing translation. All mice used for the RiboTag data were derived from the microglial RiboTag mouse background.

Data are presented as the Fold Change (FC) of Reads Per Kilobase per million mapped reads (RPKM), a standard normalization method used in RNA-seq. Old male and female mice (24 months old) were compared to young, sex-matched mice (3 months old). The amyloidosis model compares 9-month-old APP/PS1 mice *vs* wild-type littermates at 9 months postnatal. For the tauopathy model, mice at postnatal day 0 (P0) received a brain injection of AAV-Tau-P301L or AAV-GFP controls at 9 months postnatal. Intraperitoneal injections of LPS (2 mg/kg) and Poly(I: C) (12 mg/kg) or saline as a control were administered in the acute inflammation model, with microglial extraction performed 24 hours post-injection. For all experiments, n=4-5 mice per treatment or model. Full methods can be retrieved from Kang *et al.* (2018) [36].

### 2.4 Aggregate Single Cell/Nuclei Sequencing of Human Microglia

The *Chan Zuckerberg (CZ) CellxGene* Discover (https://cellxgene.cziscience.com/) gene expression platform was assessed [27] to analyze human microglial cells in non-diseased (“normal”), AD, and from 19 published and peer-reviewed datasets [29, 37-54], 1 pre-print publication [55], and a small unpublished dataset. Dot plots are created *via* the platform, which normalizes data with a log transformation of scaled pseudocounts (ln(CPTT+1)) followed by averaging. Cells that display low coverage (less than 500 genes expressed) are excluded from analysis and are not included in the study of percent cells expressing a given transcript. The platform recognizes that data are normalized and concatenated, but not integrated. There may still be significant batch effects present in this data. To aggregate the data used in the current publication, the “Gene Expression” platform of CZ CellxGene was used and filtered by disease (“normal”, “Alzheimer disease” (sic)), tissue (“brain”), organism (“Homo sapiens”), and grouped data separately based on “Publication” and “disease”. Cell type chosen for the analysis was “microglial cell” (Cell Ontology ID: CL:0000129) and “mature microglial cell” (Cell Ontology ID: CL:0002629). Canonical human microglial markers were computationally derived from *CZ CellxGene: Discover Census* and Disease Associated and Neurodegenerative microglia (DAMs and MgnD) markers are from [44, 45].

### 2.5 Differential Gene Analysis on Human Microglia from Different Severities of Alzheimer’s Disease

The Alzheimer’s Cell Atlas (TACA- https://taca.lerner.ccf.org/) [28] was utilized to assess whether Nogo-family gene expression was significantly influenced by the severity of AD or the sex of the patient. Two datasets were available with distinct cell type segregation within database analysis capabilities [29, 30]. The differential gene expression analysis (DEG) analysis was performed on both single-nuclei sequencing data of microglia and other cell types derived from entorhinal cortices. The first dataset analyzed consists of tissue grouped according to Braak stage (0, II, or VI). Nuclei were extracted from human (males) entorhinal cortex tissue: n=3 Braak stage 0 patients (ages 60, 50, and 71), n=4 Braak stage II patients (ages 72, 87, 91, and 77), and n=3 Braak stage VI patients. The original data are from Leng *et al*. (2021) [29]. No confounding adjustment was done in analysis. The other dataset consisted of both male and female AD and control subjects (AD: male, n=2; female, n=1; Control: male, n=2; female, n=1). Data were analyzed by group (Female *vs.* Male), sex across disease states, and by disease state with adjustment for sex [30]. Volcano plots were visualized by calculating the -log_10_(p-adjusted value) of each gene and plotting it against the log2 fold change of that gene. DEGs were directly downloaded from the “Differential Expression” portal of each dataset on the *TACA* website.

### 2.6 Data Analysis and Visualization

Visualizations were generated using the original publications’ datasets, with analyses conducted using GraphPad Prism V10, and R Studio V4.4.1. For the R-Studio-generated heatmap visualizing microglial expression of Nogo-family genes from E17 to P60, genes were grouped and expression levels normalized to their maximum expression over the examined time points. This normalization enabled the comparison of gene expression patterns across different time points, providing a visual representation that is readily interpretable. Other heatmaps were created using Prism V10. For single cell/nuclei data visualization, we used the *CZ CellxGene Discover* gene expression platform.

## 3 RESULTS

### 3.1 Nogo-Signaling Family Members are Expressed Differentially by Cell Type

Previous work has described regional differences in Nogo-family member gene expression in the mouse brain from young to old animals using *in situ* hybridization [56] in addition to region specific changes of these genes following neuroexcitatory stimulation (cocaine and kainic acid) [7]. Significant regional differences in expression were observed across both the mouse lifespan and following stimulant challenges in adult mice, indicating a dynamic gene family that may have implications in pathological contexts. The distribution of Nogo-related proteins throughout subpopulations of cells within the brain may help contextualize the understanding of the diverse roles that these receptors and ligands play in both chronic neurodegenerative disorders and acute injuries, such as Spinal Cord Injury (SCI), Traumatic Brain Injury (TBI), or stroke.

**Fig. (3):**
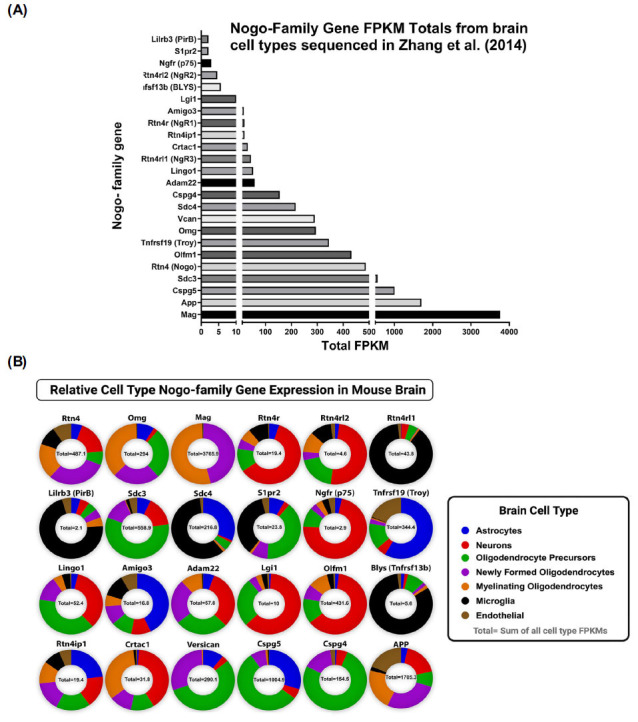
Nogo-family gene expression in a variety of brain cells. Data retrieved from Zhang *et al*. (2014) shows a wide variety of cell-type specific expression of Nogo-related genes. (**A**) Magnitude of total sum of FPKM contributions of all cells (astrocytes, neurons, oligodendrocyte precursors, newly formed oligodendrocytes, myelinating oligodendrocytes, microglia, and endothelial cells). (**B**) The “total” FPKM contribution of all cell types is shown in the center of each donut graph with the fraction of each cell-specific expression represented proportionately as a shaded region of each ring. If differing, gene abbreviation-protein abbreviation: *Rtn4*-Nogo; *Omg*-OMgp; *Rtn4r*-NgR1; *Rtn4rl2*-NgR2 (Nogo receptor 2); *Rtn4rl1*-NgR3 (Nogo receptor 3); *Lilrb3*-PirB; *Ngfr*-p75; *Tnfrsf19*-Troy; *Tnfrsf13b*-Blys; *Rtn4ip1*-Reticulon 4 interacting protein 1.

The BrainRNAseq database (www.brainrnaseq.org) was employed [31] to assess Nogo family member expression in the different cell types of the brain (astrocytes, neurons, oligodendrocyte precursors, newly formed oligodendrocytes, myelinating oligodendrocytes, microglia, and endothelial cells). As shown in Fig. (**3**), Nogo-related gene expression spans several orders of magnitude in expression differences (Fig. **3A**) across diverse brain cell types (Fig. **3B**) with varied levels of expression throughout different cell types. Fig. (**3A**) displays the magnitude of expression of each transcript visualized as a sum of each cell type’s Fragments Per Kilobase of transcript per Million mapped reads (FPKM), which is a standard expression level normalization method for RNAseq data. Fig. (**3B**) displays donut graphs of the relative FPKM counts percentage of total FPKM (Fig. **3A**) from all cells’ contributions. This display was chosen to quickly visualize which cell types have the highest expression levels of particular genes. *Rtn4* (Nogo), *Rtn4rl1* (NgR3), Sdc4 (Syndecan 4), and *Tnfrsf13b* (Blys) are genes in the Nogo family that are most highly enriched in microglia (black), with *Rtn4r* (NgR1) and *Lingo1* also showing expression to lesser extents. This data aligns with evidence that microglia express NgR1 and respond to NgR1 ligands by exhibiting impaired mobility/adhesion capabilities, as well as increased inflammatory signaling [15, 57-59]. Data are available in Supplemental File **1**.

### 3.2 Nogo-Family Gene Expression from Embryonic Stages to Old Age Shows Varied Expression and Sex Differences

**Fig. (4):**
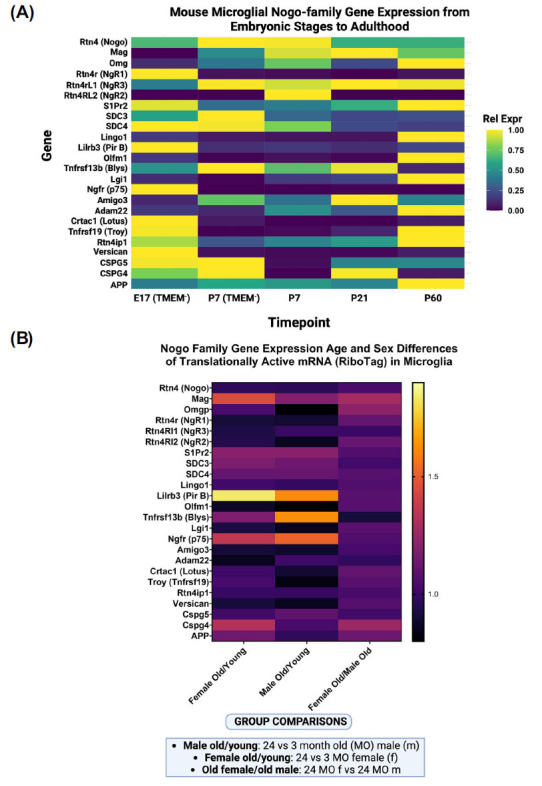
Sex and age related differences in Nogo-family gene expression in mouse microglia. Data retrieved from the studies of Bennett *et al.*, (2016) (**Α**) and Kang *et al*., (2018) (**B**). RNAseq data shows age related expression of many of the Nogo-family genes in microglia, with notable elevations in many genes at E17 and P60 (**A**). Translationally active mRNAs from microglia in 24-month-old female and male mice show elevated levels of several genes compared to 3-month-old (young) mice (**B**). Fold change values are reflected in the scale to the right of the heatmap.

Mouse microglia cells undergo a wide variety of transcriptional changes over short periods of time from their immature states during embryonic development to their mature postnatal states one week following birth and into adulthood [60]. To investigate whether Nogo-family gene expression is involved in the differing maturation states of microglia, data from www.brainrnaseq.org were utilized, which provides information on mouse microglia from embryonic day 17 (E17) to postnatal day 60 (P60) [32]. Transmembrane protein 119 (*Tmem119*) is a canonical marker specific to microglia but does not appear until microglia become mature. A mixed population of *Tmem119*^+^ and *Tmem119*^-^ microglia is present at postnatal day 7, indicating that microglial niche maturation occurs at this stage of development. Twenty-four Nogo-family genes were probed and it was found that a subset had their highest levels of expression at E17 (*Rtn4r, S1pr2, Lilrb3* (PirB), *Ngfr* (p75), *Crtac1* (Lotus), Troy, and Versican), while a different subset had highest expression levels at P60 (*Mag*, Syndecan 4 (*Sdc4*), *Lingo1*, *Olfm1, Lgl1, Tnfrs13b* (BLYS), *Adam22*, and *App*) (Fig. **4A**). *Rtn4ip* stands out as the only gene with high expression levels at both E17 and P60. Full data can be viewed in Supplemental File **2**. At E17, receptors and coreceptors for the Nogo-signaling system are most active (*Rtn4r, S1Pr2, Lilrb3, Crtac1*, and *Ngfr*) with other genes most highly expressed in adulthood (*Mag, Sdc4, Lingo, Olfm, Blys, Lgi1, Adam22*, and *Rtn4ip1*).

Around E17, mouse microglia are highly phagocytic and actively shaping stable neuronal circuits [61]. Previous studies have shown developmental regulation of *Rtn4r* (NgR1) [62, 63]. *Rtn4* (Nogo) is most highly expressed in microglia at P7, mirroring expression of Nogo in oligodendrocytes at similar postnatal ages [62, 64, 65]. Previous work using *in situ* hybridization to assess spatial differences in the expression of Nogo-family genes has shown similar global patterns of receptor and co-receptor expression during the first two postnatal weeks, with Nogo expression also found to be highest during this same time period [56].

Smedfors *et al.* (2018) [56] have provided the most comprehensive examination of expression differences of Nogo-family genes from birth to old age (24 months). However, sex differences in the expression of these genes in old and young mice have not been well described. In Fig. (**4B**), translational profiling data from Kang *et al*. (2018) [36] is presented to observe these differences. RNA-seq data has been derived from microglial mRNA that is undergoing active translation at the time of extraction (as described in Fig. **2**), providing a more nuanced examination of which transcripts may be necessary at a given time. Microglia from 24-month-old females compared to 3-month-old females show an elevation of several transcripts, including *Mag* (1.44-fold), *S1Pr2* (1.2-fold), *Ngfr* (1.36-fold), Lilrb3 (1.83-fold), and *Cspg4* (1.34-fold). Most other transcripts remain stable. Old male mice, compared to young mice, show similar elevations of *Mag* (1.19-fold), *Ngfr* (1.51-fold), and *LilrB3* (1.62-fold. Notably, they show decreases compared to young males across far more transcripts than females and their younger counterparts. These include *Omg* (0.81-fold), *Olfm* (0.81-fold), *Troy* (0.83-fold), *Lgi1* (0.86-fold), *Rtn4RL2* (0.86-fold), and *Rtn4r* (0.89-fold). Old female mice show higher expression of *Mag* (1.28-fold), *Omg* (1.22-fold), *Rtn4r* (1.11-fold), *Crtac1* (1.10-fold), *Cspg4* (1.26-fold), and *APP* (1.13-fold) than old males. All transcripts except *Blys* (0.91-fold) are elevated in older females compared to males. Complete data are provided in Supplemental File **3**.

### 3.3 Nogo-Family Gene Expression is Regulated in Microglia During Acute and Chronic Inflammation Models

**Fig. (5):**
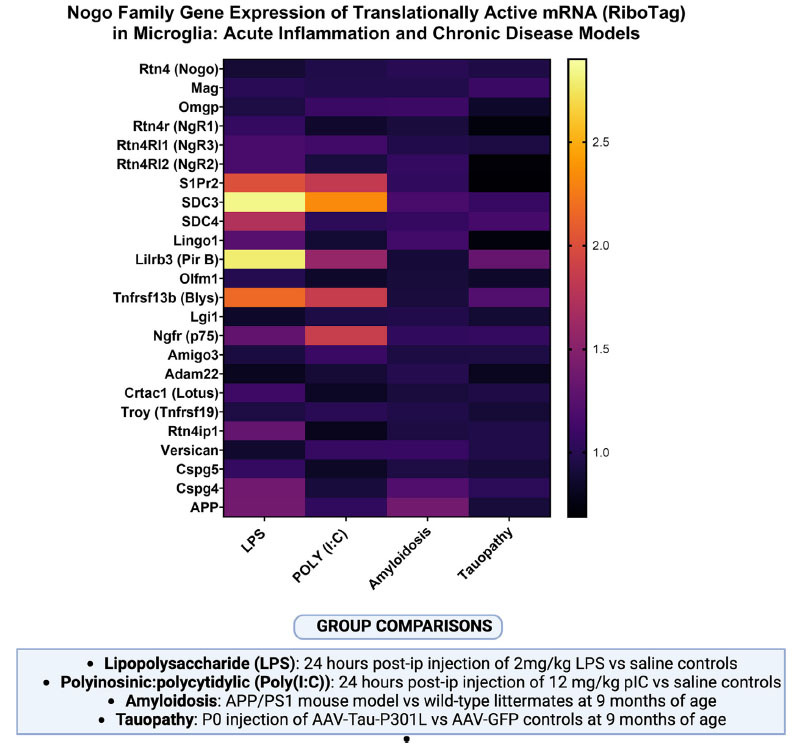
Nogo-family gene expression regulation in microglia following acute inflammatory challenges and chronic disease models. Translationally active mRNA was extracted from mouse microglia 24 hours after IP injection of LPS of POLY (I:C) or in transgenic APP/PS1 mice (amyloidosis) and mice with viral induced overexpression of Tau protein (tauopathy). Nogo-like signaling gene patterns in relation to sham injected animals are shown; n=3-4/group. Fold changes are in relation to RPKM expression levels, with scale for FC shown to the right. Data retrieved from Kang *et al*., (2018).

This study investigated whether the expression of Nogo-family genes in microglia is regulated in response to injury or disease. As microglia are highly dynamic in their ability to change transcriptional programs in response to aberrant brain activity or damage, we utilized RiboTag data from Kang *et al.* (2018) [36] which includes translationally relevant microglial gene expression following LPS or *POLY* (I: C) challenges and models of AD (amyloidosis and tauopathy) in nine-month-old mice compared to control mice (saline controls and wildtype age matched littermates). Common *in vivo* assays for neuroinflammation utilize Intraperitoneal (IP) delivery of LPS or poly(I:C), which initiate an inflammatory response *via* their respective activation of TLR4 or TLR3, and the downstream master transcriptional regulator NF-κB [66]. Kang *et al*. (2018) [36] applied these models to investigate transcriptional changes in microglia following a systemically induced inflammatory insult. Although applied to activate microglia globally (functionally agnostic), the multiple activation states of these cells are not reflected in the RNA-seq results. It can be observed, however, that actively translated *S1pr2* (2-fold), *Sdc3* (2.84-fold), *Sdc4* (1.73-fold), *Lilrb3* (2.79-fold), and *Tnfrs13b* (2.16-fold) are upregulated during the inflammatory insults (2 mg/kg LPS or 12 mg/kg POLY (I: C)), whereas downregulation of Adam22 (-1.24-fold), *Crtac1*, *Lgi1*, and, to a smaller extent, *NgR1* are elicited (Fig. **5**). Interestingly, expression levels of Nogo-family genes in the amyloidosis model were relatively stable, as compared to wildtype controls. In contrast, in the tauopathy model, most Nogo-family genes were downregulated (*Omg: 0.85-fold; Rtn4r: 0.73-fold; Rtn4rl2: 0.71-fold; Lingo1: 0.73-fold; Lgi1: 0.89-fold; Adam22: 0.81-fold; Crtac1: 0.95-fold; Cspg5: 0.91 fold; APP: 0.91-fold*). All data are viewable in Supplemental File **3**.

### 3.4 Aggregate Single Cell/Nuclei Sequencing Data from Microglia in Human AD Patients Reveals *LINGO1* Enrichment in AD Microglia

**Fig. (6):**
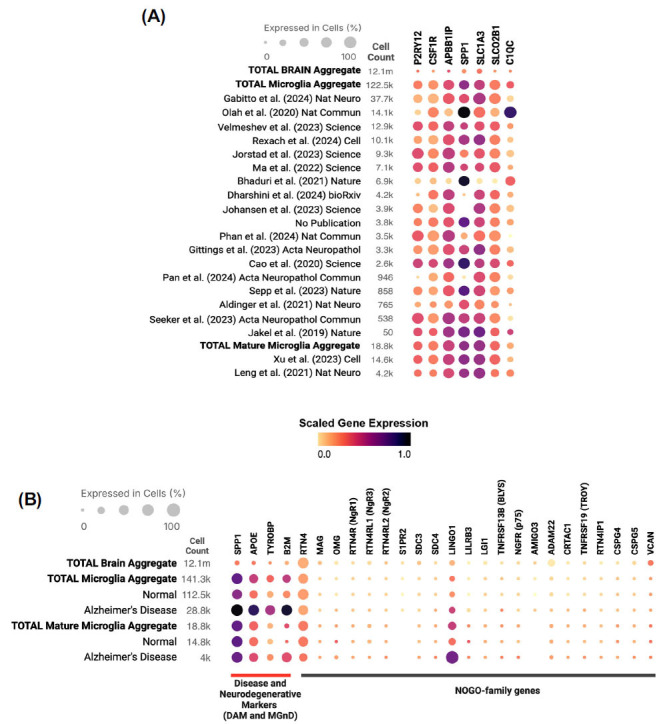
Aggregate single cell/nuclei sequencing of microglia from human AD patients using the CZ CellxGene Discover Census. The census can computationally determine scaled expression levels of genes across many publications/datasets. Here, canonical microglial markers are presented from all datasets analyzed (**A**) and Nogo-family genes in relation to disease state. These 21 datasets include 12.1 million brain cells and 141.3K microglia aggregated. Total cell counts from each publication are included to the left of the dot plot. All data from these studies were summarized by disease in the CZ CellxGene Discover Gene expression portal (**B**). Total brain microglia and mature microglia expression levels are shown in bold text of **B**, with “disease” state nested underneath. Microglia and mature microglia from the AD datasets are highly enriched for DAM and MGnD gene signatures. All microglia datasets show similar levels of *RTN4* expression, while *LINGO1* is potently upregulated in the AD datasets. Other Nogo-family genes are minimally or not expressed in human microglia.

The CZ CellxGene Discover platform was utilized as a platform to aggregate 21 human brain single-cell sequencing datasets specific to AD to assess whether Nogo-family genes were differentially regulated in this condition (Fig. **6**). Across these 21 datasets, consistent expression of canonical microglial markers, including *P2Ry12, Csf1R, APBB1IP, SPP1, SLC1A3, SLC102B1*, and *C1QC* was observed (Fig. **6A**). Although there are some discrepancies with expression levels in some publications, these datasets include microglia from diseased brains which affects canonical marker expression depending on severity or stage of disease from patient brain samples. This is particularly true for the gene *P2RY12*, which is known to drastically reduce expression in the presence of proinflammatory pathology [67, 68]. Data related to Fig. (**6A**) can be reviewed in Supplemental File **4**.

Subsequently, this study investigated Nogo-family gene expression in relation to Disease Associated Microglia (DAM) and Neurodegenerative Microglia (MGnD) gene profiles (Fig. **6B**), previously established in mouse models of AD [69, 70]. In humans, these microglial gene signatures are prevalent and highly enriched in AD patients, with *B2M*, *APOE*, and *B2M* expressed in over 65% of microglia at high levels, and *TYROBP* expressed in 55% of cells.

Of the Nogo-family genes, only *RTN4* (Nogo) is stably expressed at medium levels across all microglia with smaller percentages of cells expressing in AD (49.44%) and larger percentages in normal (control) microglia (59.55%). While *LINGO1* stands out as highly expressed in large numbers (85.06%) of AD-associated mature microglia (Cell Ontology (CL):0002629), other Nogo-family genes are minimally or not expressed in human microglia. Data are available in Supplemental File **5**.

### 3.5 The Alzheimer’s Disease Cell Atlas (TACA): Non-Biased Differential Gene Expression Analysis Using Two Separate Datasets Shows Opposite Expression Patterns of *LINGO1*

As this study has described microglial enrichment of *LINGO1* in human AD (Fig. **6**), it also investigated whether the severity of the disease affected Nogo-family gene expression. *The Alzheimer’s Cell Atlas* (TACA) was assessed [28] and used droplet single cell nucleus sequencing data from human brain entorhinal cortices [29] to specifically interrogate differentially expressed genes (DEGs) in microglia from male patients with AD Braak stage 0 (no disease) to Braak stages II and VI, with additional comparison of Braak stages II to VI. The Braak staging system describes the progressive deterioration of the entorhinal cortex, which is typical in cases of AD and other neurodegenerative disorders. The larger the Braak stage number, the more severe the disease has progressed to different regions of the brain. Neurofibrillary tangles and neuronal deterioration first appear in the entorhinal cortex (Braak stages I and II), with the disease eventually progressing to the hippocampus and limbic structures (Braak stages III and IV). Braak stages V and VI are the most severe stages of the disease, with broad exposure and damage evident within the neocortex [71].

**Fig. (7):**
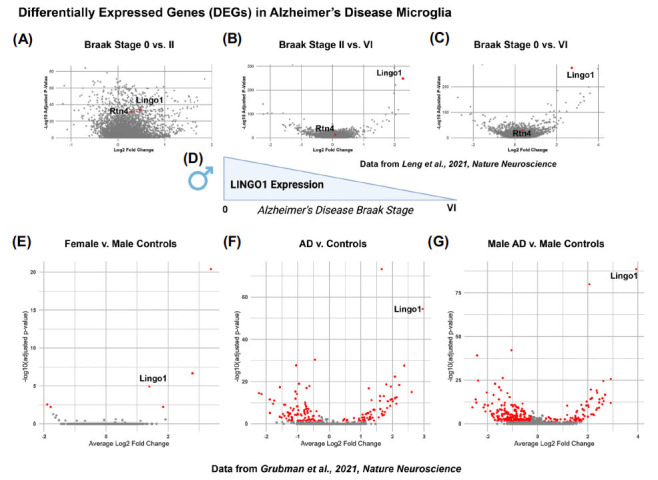
Comparison of differentially expressed entorhinal cortex-derived human microglial genes between AD or control patients. A-C describes data from Leng *et al*. (2021) in patients with Braak stage 0, 2, or 6 AD. This dataset shows that as AD progresses, microglial enrichment of *LINGO1* decreases and is a top DEG earlier in the development of AD, while *RTN4* also marginally upregulated in earlier AD stages (**A-C**). (**D**) Visualization of *LINGO1* expression during progressively severe AD as shown in Leng *et al*. (2021) (n=3 or 4 for each group). (**E**-**F**) Data from Grubman *et al*. (2019) shows the opposite expression pattern of *LINGO1* in microglia from AD patients *vs.* Control patients. *LINGO1* is a top DEG in microglia from the female control patient (n=1) compared to the male controls (n=2) (**E**). When adjusted for sex, microglia from AD patients (n=3) are significantly enriched with *LINGO1* expression (**F**). (**G**) Males in the AD cohort (n=2) show *LINGO1* as the top DEG in microglia compared to the male controls (n=2). Genes highlighted in red are considered significant DEGs (*p*<0.05 and log_2_FC > 0.25).

Interestingly, a decrease in *LINGO1* and *RTN4* expression was observed at higher Braak stages, as reported by Leng *et al.* (2021) [29]. Comparing Braak stages 0 (control) to II (Fig. **7A**), *LINGO1* is 291^st^ top Differentially Expressed Gene (DEG) in microglia, with a 0.46 log_2_ Fold Change (FC) difference in expression (*P value_adj*= 2.42E^-34^). *LINGO1* expression was observed in 77% of Braak stage 0 microglia and 92% of Braak stage II microglia. *RTN4* is the top 338^th^ significant DEG, with an expression of 0.28 log2FC, and was detected in 13% and 28.5% of all microglia in Braak stages 0 and II, respectively (*P* value_adj = 3.54E^-32^). Between Braak stages II and VI (Fig. **7B**), *LINGO1* is a top 3 DEG, showing 2.26 log_2_ FC higher expression in the earlier stages of symptomatic AD (*P value_adj*= 6.54E^-249^), while *RTN4* moves down to the 797^th^ top DEG (expression 0.09 log_2_FC) (*P value_adj*= 6.68E^-14^). Comparison of microglia from patients with Braak stages 0 (control) *vs.* VI shows *LINGO1* as a top 2 upregulated DEG (2.78 log_2_FC; *P value_adj*= 5.53E^-275^) (Fig. **7C**). *RTN4* is the 370^th^ top DEG (0.38 log_2_FC; *P value_adj=* 1.95E^-2^*^9^)*. Interestingly, this pattern of *LINGO1* downregulation in progressive male AD patients was also observed in other cell types analyzed within the dataset, including astrocytes, inhibitory and excitatory neurons, endothelial cells, Oligodendrocyte Precursor Cells (OPCs), and oligodendrocytes. *LINGO1* is a top 5 significantly upregulated gene among the earlier stages of male AD in most cell types analyzed (volcano plots for all cell types and comparisons are provided in Fig. **S1** and **S2**). A visualization of *LINGO1* expression related to Braak stages in males is shown to make the dataset interpretation more straightforward (Fig. **7D**). Full DEG analysis data can be downloaded and accessed here: https://taca.lerner.ccf.org/e/dataset/GSE147528_EC/.

To determine whether sex differences and AD diagnosis affected *LINGO1* gene expression, an additional dataset available on the *TACA* platform from Grubman *et al*. was used (2019) [30]. Upon initial examination of the data, *LINGO1* emerged as a top significantly expressed DEG (#3, 2.784 log_2_FC; *P value_adj=*0.00001) in microglia from the female control patient compared to the male control patients (Fig. **7E**). Amongst the control patients, the female individual expressed *LINGO1* as a top significant DEG in other cell types, including neurons, astrocytes, oligodendrocytes, and OPCs. Additionally, in microglia from AD *vs.* control patients (adjusted for sex), *LINGO1* is the second highest significant DEG (log_2_FC= 2.956; *P value_adj*=4E^-55^) (Fig. **7F**). Although the female control patient showed significantly higher *LINGO1* expression than the female AD patient, *LINGO1* does not appear in any cell type as a significant DEG when comparing the disease states of the female patients (data not shown). However, when comparing male AD patients to male control patients, *LINGO1* is the top significant DEG in microglia (Fig. **7G**) (log_2_FC= 3.93, *P value adj*=3E^-89^), neurons, oligodendrocytes, OPCs, astrocytes, and endothelial cells. Volcano plots for all cell types and comparisons described in Fig. **7E**-**G** are shown in Fig. **S3** and **S4**. Complete data sets of comparisons summarized here can be accessed at https://taca.lerner.ccf.org/e/dataset/GSE138852/. The Grubman *et al.* (2019) [30] dataset is summarized in Fig. (**8**).

## 4 DISCUSSION

The current study is the first comprehensive analysis of mouse and human microglial Nogo-family gene expression. By utilizing the wealth of publicly available data, this approach assesses peer-reviewed studies that include diverse methods (standard RNAseq, RiboTag Translational Profiling, aggregate single cell sequencing, and differential gene analysis) and model systems (LPS, Poly (I: C), and transgenic (Tg) mice) to interrogate the line of inquiry. Two new resources were used to analyze human microglia. 1) the *Chan Zuckerberg (CZ) CellxGene Discover* platform [27] to aggregate 21 publicly available single cell/nuclei sequencing datasets from human AD and control patients and 2) *The Alzheimer’s Cell Atlas (TACA)* [28] to perform differential gene analysis on microglia and other cells types based upon sex, disease state, and disease severity. This represents a new approach to examining broad swaths of single-cell or nuclear data on user-friendly platforms. In mice, *Rtn4, Rtn4rl1* (NgR3), *Mag, S1pr2*, syndecan-3 (*Sdc3*), syndecan-4 (*Sdc4*), and *Lilrb3* (PirB) were found to be enriched in mouse microglia relative to other brain cell types. In humans, microglial enrichment of *RTN4* and *LINGO1* in AD microglia was identified through analysis of an aggregate single-cell dataset. To further investigate this finding, differential gene analysis was performed on two individual single-cell datasets of microglia and other cell types from the entorhinal cortex. From one study [29], it has been found that male AD patients with increasing severity of AD have significant downregulation of *RTN4* and *LINGO1*. The other study [30] has shown the opposite relationship between males with AD and control patients and an overall elevation of *LINGO1* in female control patients compared to males. These datasets both show *LINGO1* to be a top significantly expressed DEG, but the importance of the gene is not evident, requiring further inquiry.

In this analysis, Nogo-family microglial gene expression from mouse embryonic stages (E17) to adulthood (P60) largely mirrored total brain spatial gene expression patterns previously investigated [7]. Microglia express increased levels of Nogo receptors and coreceptors for the Nogo-signaling system early in development (E17) (*RTN4R, S1Pr2, Lilrb3, Crtac1*, and *Ngfr*) with other genes most highly expressed in adulthood (P60) (*Mag, Sdc4, Lingo, Olfm, Blys, Lgi1, Adam22*, and *Rtn4ip1*), and *Rtn4* most highly expressed in adolescence (P7). This study evaluated whether these baseline levels of expression data are regulated further by sex, in old age (24 months), or during acute injury [LPS or Poly (I: C) and in models of chronic neurodegenerative conditions (APP/PS1 and Tau overexpressing mice)] using RiboTag data from Kang *et al*. (2018) [36]. This dataset provides gene expression data only from mRNA that is actively being translated during the time of microglial extraction. The authors of this study broadly recognized similar gene expression patterns between old age and the acute injury models with almost no overlap in the chronic neurodegenerative models. It was similarly found that overlap existed between old age and acute injury models, with *S1pr2, Lilrb3, Tnfsf13b,* and *Ngfr* being upregulated in both datasets. Nogo-family genes *S1pr2, Sdc3, Sdc4, Lilrb3, Tnfsf13b,* and *Ngfr* are upregulated compared to saline injected controls, while other genes remain stably expressed. Interestingly, microglia from 9-month-old APP/PS1 mice do not show differentially regulated Nogo-family genes compared to wild type age matched littermates (controls), while the 9-month-old Tau overexpressing mice display broad downregulation of most Nogo-family genes compared to control mice. Sex and age appear to influence levels of expression of Nogo-family genes, as 24-month-old female mice have elevated levels of nearly all genes probed, as compared to 3-month-old young females and 24-month-old males. Human females have a higher propensity for AD, and these findings raised the question as to whether Nogo-family gene enrichment in microglia may be a contributing factor in human disease.

The focus of this study on microglial Nogo-family genes as potential mediators of microglial reactivity is supported by a variety of recent studies, which include reducing activity of Nogo-A [18, 72, 73] or *LINGO1* [19, 74, 75] with neutralizing antibodies, shRNAs, antagonists, or Knock Out (KO) or Knock Down (KD) Tg mice that effectively reduce inflammation in a variety of rodent models. Nogo-A was shown to play a critical role in the regulation of pro-inflammatory genes [76], with Nogo-B similarly facilitating LPS-induced increases in pro-inflammatory cytokine production [77]. In a separate *in vitro* LPS model using PC12 cells, silencing Nogo-A *via* RNA interference downregulated TNF-α and IL-6 protein release. Additionally, it blocked reductions in tyrosine hydroxylase, a common feature of PD [78].

Blockade of Nogo-A, NgR1, and other related proteins has been shown to enhance synaptic plasticity and result in behavioral improvements across a broad range of CNS injury models. This has been demonstrated by the use of neutralizing antibodies [79, 80], small interfering RNAs [73], soluble receptor fragments [81], peptide antagonists [82], gene KO or KD [83], as well as blockade of the RhoA or ROCK signaling cascades [84]. All these approaches enhance the regenerative sprouting and growth of lesioned neuronal fibers as well as the compensatory collateral sprouting of intact fibers in models of spinal cord or brain injury. The wide variety of studies that have used germline KO of Nogo ligands and receptors [83, 85, 86] provide insights into total expression contributions of these proteins to phenotypes; however, outcomes of injury models are likely more nuanced when KO/ KD or overexpression are undertaken in specific cell types. For example, preliminary mouse studies of Nogo-A KO in oligodendrocytes and neurons affect dendritic arborization and spine density differentially [87] with oligodendrocyte KO more beneficial to SCI recovery than neuronal KO [88]. These studies have highlighted cell-type-specific contributions of the Nogo-family genes and potential outcomes for treatment, hence the importance of the current research evaluating microglial gene expression.

It has been shown that there are diverse expression patterns of Nogo-family genes in mouse and, to a lesser extent, in human microglia. *RTN4* and especially Lingo-1 appear to be clinically relevant targets for AD [89], and are highly enriched in human microglia from AD patients according to the aggregate and DEG single-cell/nuclei data analyses presented herein. Preclinical Tg AD animal models demonstrate significant improvements in neuron preservation and cognitive deficits using Lingo-1 [19, 20, 90] and Nogo-A (Rtn4) [91, 92] blocking treatments. This study indicates that old (24 months) female rodents have overall higher levels of Nogo-family genes than old males. A comprehensive study on microglia throughout the lifespan of a mouse, using single-cell sequencing, concluded that mouse microglia exhibit no sex differences in transcriptional signatures [60]. However, others have reported sex-specific features of microglia [93, 94].

The rodent studies analyzed herein indicate that old females have higher Nogo-family genes than males; however, our DEG analysis of human tissue reveals the potential importance of *RTN4* and *LINGO1* in both male AD patients and healthy females. Although Grubman *et al*. (2019) [30] did not evaluate a large number of patients, the findings align with those of other researchers [22]. Belonwu *et al.* (2021) [23] analyzed the data from Grubman *et al*. (2019) [30], along with single-cell sequencing of AD patient samples from Mathys *et al*. (2019) [22], and noted a similar overall upregulation of *LINGO1*, but only in female AD patients compared to controls. Across cell types, Belonwu and colleagues do note significant upregulation of *LINGO1* in male AD samples. This independent analysis yielded similar findings as this study, further pointing to the potential for *LINGO1* as a drug target for AD. The higher propensity for AD diagnosis in females has typically been attributed to increased longevity of women, though this is not a definitive explanation [95]. These findings of significant differences in *LINGO1* expression in the control female patient compared to male controls [30] are interesting and warrant further investigation.

Patients with temporal lobe epilepsy [96], multiple sclerosis [97], schizophrenia [98], AD [99], and PD [100] show elevated levels of Nogo-A in nervous system tissue. Nogo-A is upregulated in AD hippocampus and is localized to amyloid plaque deposits and reactive glial cells. Likewise, NgR1 expression is elevated in the CA1 and CA2 regions of human AD hippocampus [101]. NgR1 is considered to have a role in amyloid-β peptide (Aβ) generation, with over-expression in AD mouse models resulting in lowered Aβ [102]. Mice lacking NgR1 demonstrate elevated Aβ accumulation [102]. However, a reduction (or deletion) of Nogo-A results in improved performance and reduced neuropathology in AD Tg mice [83]. Conversely, overexpression of NgR1 in forebrain neurons in AD Tg mice impairs spatial cognition without influencing amyloid plaque formation [103]. In rats, Nogo-family proteins NgR1 and PirB are responsive to chronic stress stimuli, with both showing elevation in a recent study. This elevation in plasticity restricting genes could be attenuated pharmacologically or with electroconvulsive therapy [104].

Proof of concept studies lowering Nogo activity have been undertaken in non-human primates, and a variety of clinical trials are ongoing for treating SCI with a Nogo receptor decoy that captures Nogo, *Mag*, and *Omg* [105]. Clinical trials for treating multiple sclerosis utilizing Lingo-1 antibodies have broadly failed over the past 10 years (NCT03222973). Interestingly, results have been mixed in preclinical studies comparing Nogo ligand and receptor knockout studies with antibody treatments [106]. Furthermore, mouse strain-specific differences in axonal recovery from SCI were observed in Nogo-A KO studies [107], although this finding has been disputed [108]. As most KO studies target all cells in the rodent body, it is vital to consider cell-type-specific contributions to observed phenotypes. As the Nogo-signaling system involves many proteins, understanding cellular localization of each may be beneficial in focusing targeted KO studies, as highlighted in the current study.

Targeting the cell-specific localization of Nogo-family genes appears to be key in understanding the pathology associated with these genes. By aggregating a wealth of data from preclinical rodent studies and clinically relevant human samples, it is highlighted that the potential roles of Nogo-family genes in injury and disease are evident. Although this is an essential step in understanding microglial contributions in the Nogo signaling system, the data we have analyzed presents some caveats. The enrichment of *RTN4* and *LINGO1* in human AD microglia does not correlate with findings from the AD mouse models we analyzed. This highlights the lack of clinical translation of animal studies that has haunted the development of effective AD treatments for humans over the past two decades [109]. Additionally, it is essential to consider regional differences in microglial gene expression, which have been observed in rodents [110-112] and humans [113-115], potentially accounting for the bias in the data presented. Although microglia from the entorhinal cortex of female control and male human AD patients present highly significant changes in *LINGO1* expression, other brain regions may not show this level of enrichment or downregulation. The opposing findings in our differential gene analysis raise more questions that require deeper analysis and investigation. The Grubman *et al*. (2019) [30] study had a small number of patients to compare, which limited the definitiveness of the results. However, future work should seek to replicate and confirm sex- and region-specific cell type gene expression differences related to AD. Lastly, small populations of microglia that have disproportional influence on pathology [60] may not be reflected in the current data analyzed. As inflammation arising from microglia is a key component of all neurodegenerative diseases and brain injury, focus on these rare cell types is paramount to the discovery of new treatments [116-118]. As the *CZ CellxGene Discover* platform continues to refine cell ontologies, the inclusion of brain regions as a categorization method may make the data gleaned from the resource even more profound. Here, possible connections between Nogo-family genes in microglia during chronic and acute inflammation-related injury/disease states are presented, and further research to understand the diverse roles of this ubiquitous and clinically relevant signaling system is proposed.

## CONCLUSION

The current study is the most comprehensive analysis of Nogo-family gene expression in mice and humans. Factors that influence Nogo-family gene expression, such as age, sex, and pathological determinants, are identified in this search, and the gap between preclinical with clinically relevant datasets is bridged to relate this vital signaling system to human disease. Human microglial enrichment of *RTN4* and *LINGO1* is potentially elusive and previously ignored targets for drug targeting in AD. Further research is needed to determine the relevance of these highly enriched genes in human AD and other CNS injuries and diseases.

## Figures and Tables

**Fig. (8):**
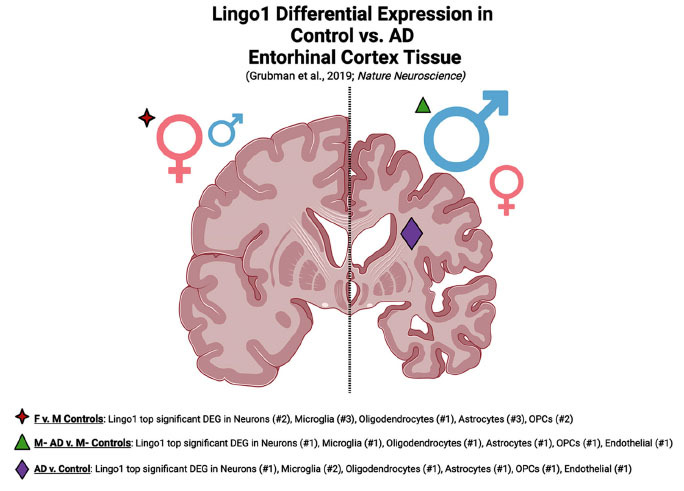
**Single cell sequencing data summary of Grubman *et al*. (2019).** This visualization emphasizes the significant differential expression of *LINGO1* in the entorhinal cortex across multiple cell types and sexes in control (left) and AD (right) patients.

## LIST OF ABBREVIATIONS

AD: Alzheimer’s Disease
BLYS: B Lymphocyte Stimulator
CCI: Controlled Cortical Impact
CL: Cell Ontology
CNS: Central Nervous System
DAM: Disease-Associated Microglia
DEG: Differentially Expressed Gene
FC: Fold Change
FPKM: Fragments per kilobase of transcript per million mapped reads
GWAS: Genome-wide Association Study
HA: Hemagglutinin
IP: Intraperitoneal
KD: Knockdown
KO: Knockout
LPS: Lipopolysaccharide
MACS: Magnetic-activated Cell Sorting
MAG: Myelin-associated Glycoprotein
Mais: Myelin-associated Inhibitors
MS: Multiple Sclerosis
NgR1: Nogo Receptor 1
Nogo: Neurite outgrowth Inhibitor
OPCs: Oligodendrocyte Precursor Cells
RhoA: Ras Homolog Gene Family Member A
ROCK: Rho-associated Coiled-coil Containing Protein Kinase
RPKM: Reads per Kilobase per Million Mapped Reads
RTN4: Reticulon 4
S1pr2: Sphingosine 1-Phosphate Receptor 2
SCI: Spinal Cord Injury
SDC4: Syndecan 4
TACA: The Alzheimer’s Cell Atlas
TBI: Traumatic Brain Injury
TLR: Toll-Like Receptor
TMEM119: Transmembrane Protein 119

## DECLARATION

Graphical abstract, Figure **2**, Figure **8**, and portions of Figures **3**-**6** were created using BioRender.com.
