# Supplementary material for: Analyses of Nogo-Family Genes in Mouse and Human Microglia Omics Datasets Identify LINGO1 as a Candidate Drug Target in Alzheimer’s Disease
Source: Curr Neuropharmacol. 2025 Jul 30;23(14):1918–38. doi: 10.2174/011570159X359944250722061312 (PMC12512228; doi:10.2174/011570159X359944250722061312)
Supplement: Supplementary file 1 [file CN-23-14-1918_SD1.pdf]

Supplementary Material

Analyses of Nogo-Family Genes in Mouse and Human Microglia Omics Datasets Identify *LINGO1* as a Candidate Drug Target in Alzheimer's Disease

Elliot J. Glotfelty[1,*], Tobias E. Karlsson[2], Luis B. Tovar-y-Romo[3], Lars Olson[2], Brandon K. Harvey[1] and Nigel H. Greig[4]

[1]Cellular Stress and Inflammation Unit, Integrative Neuroscience Department, National Institute on Drug Abuse, National Institutes of Health, Baltimore, Maryland 21224, USA; [2]Department of Neuroscience, Karolinska Institutet, Stockholm, Sweden; [3]Division of Neuroscience, Institute of Cellular Physiology, Universidad Nacional Autónoma de México, Mexico City, Mexico; [4]Drug Design & Development Section, Translational Gerontology Branch, Intramural Research Program National Institute on Aging, NIH, Baltimore, MD 21224, United States

1875-6190/25 $65.00+.00 **© 2025 Bentham Science Publishers**

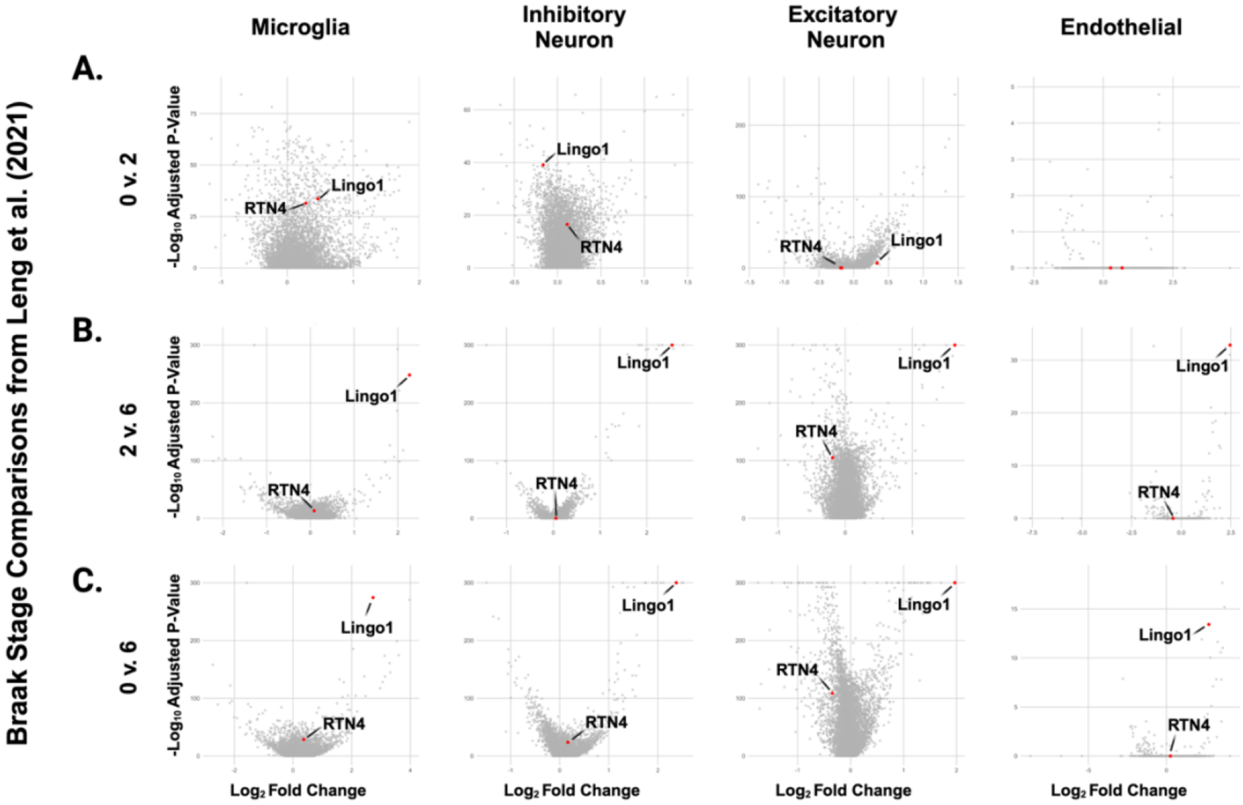

Figure S1. *Leng et al. (2021) data summary comparing DEGs across different Braak stages of AD; (A) 0 v. 2, (B) 2 v. 6, (C) 0 v. 6. DEG gene for these comparisons is shown in microglia, inhibitory and excitatory neurons, and endothelial cells.*

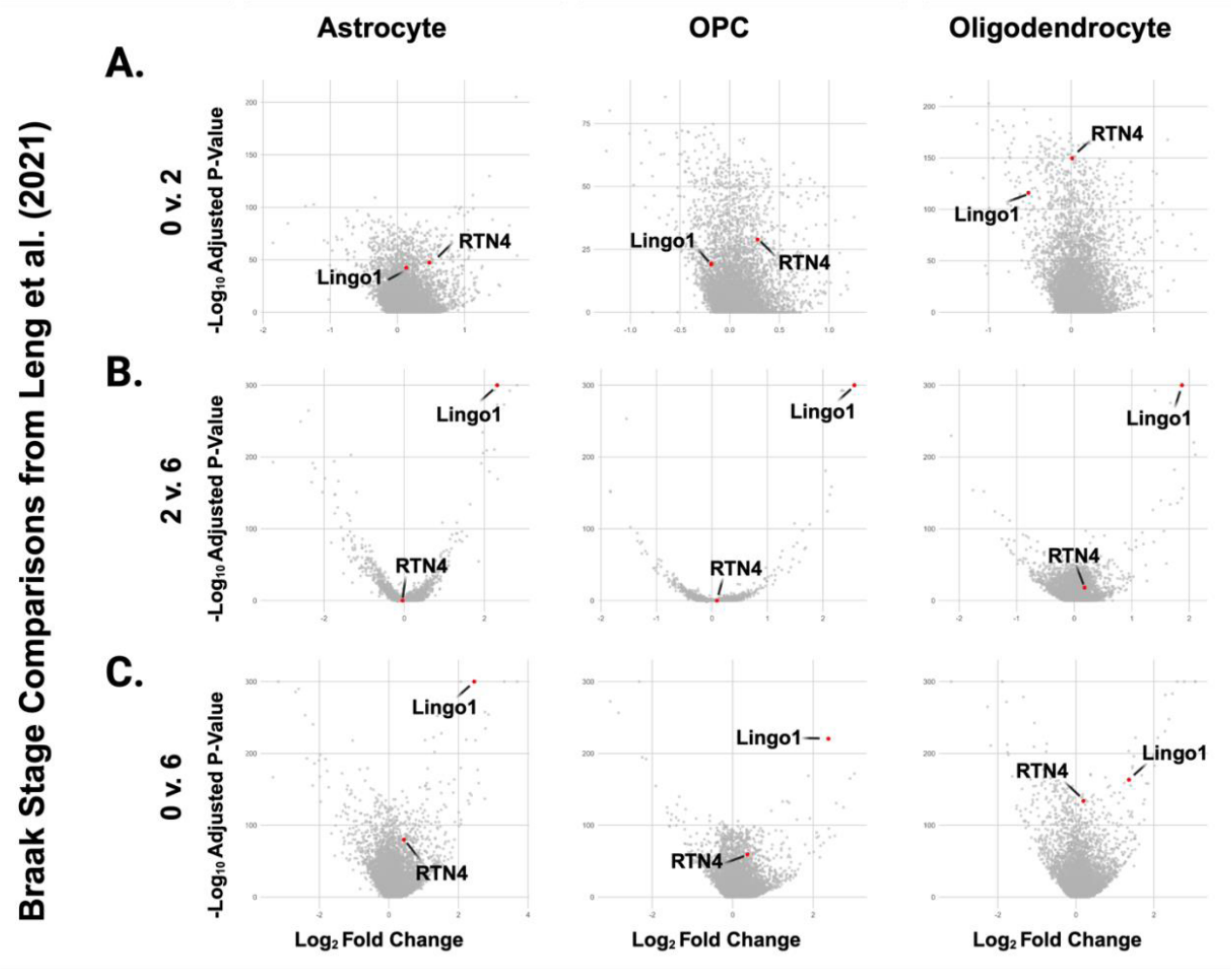

Figure S2. *Leng et al. (2021) data summary comparing DEGs across different Braak stages of AD; (A) 0 v. 2, (B) 2 v. 6, (C) 0 v. 6. DEG gene for these comparisons is shown in astrocytes, OPCs, and oligodendrocytes.*

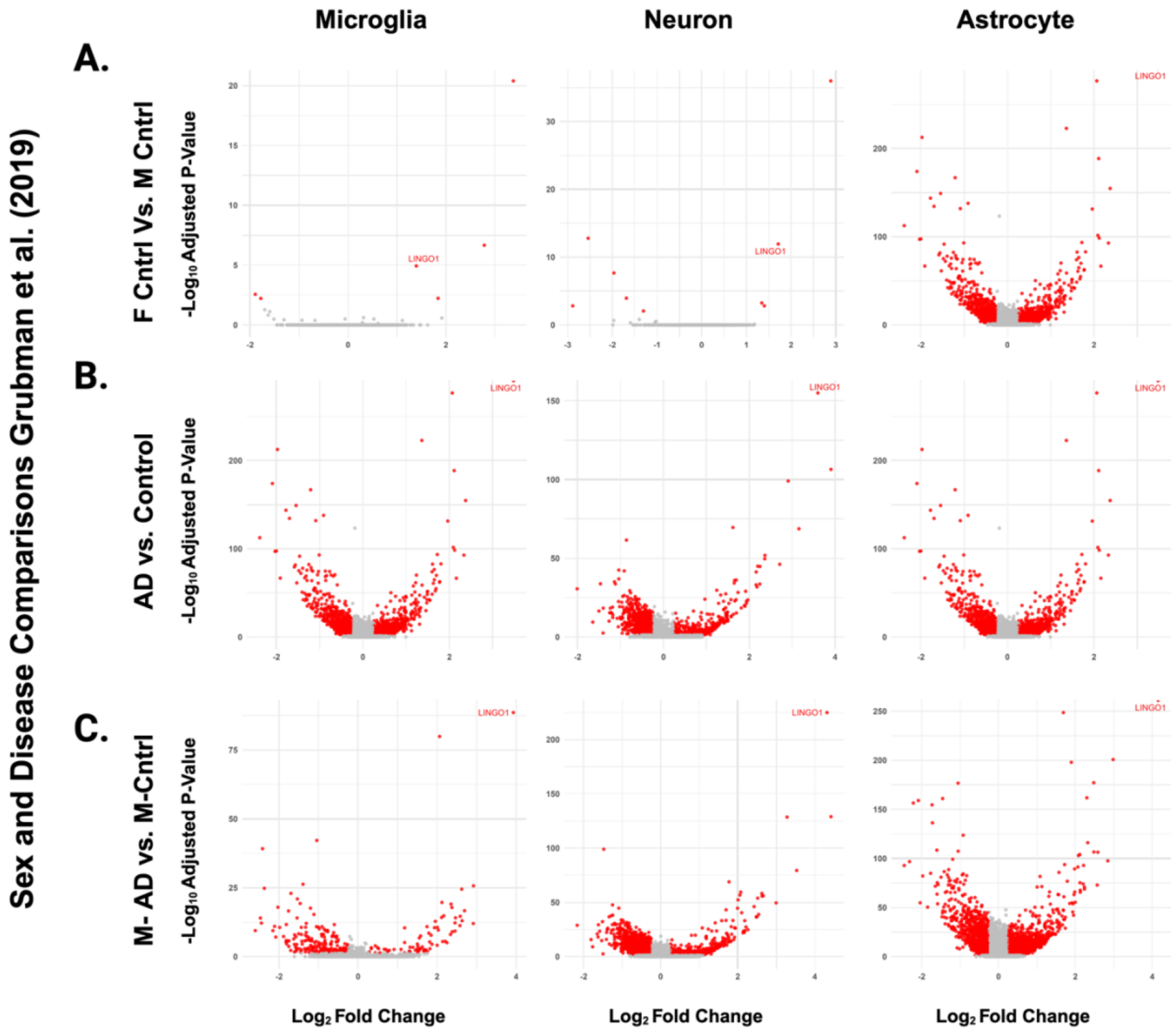

Figure S3. *Grubman et al. (2019) data summary comparing DEGs from cells in the entorhinal cortext across sex, disease state, and AD diagnosis; (A) Female Control (n=1) v. Male Control patients (n=2), (B) AD patients (n=3) vs. Control patients (n=3) (C) Male AD (n=2) v. Male Control (n=2). DEG gene for these comparisons is shown in microglia, neurons, and astrocytes. Lingo1 appears as a top significant DEG across all cell types and conditions shown. Genes highlighted in red* are considered significant DEGs *($p<0.05$ and $log_2FC>0.25$).*

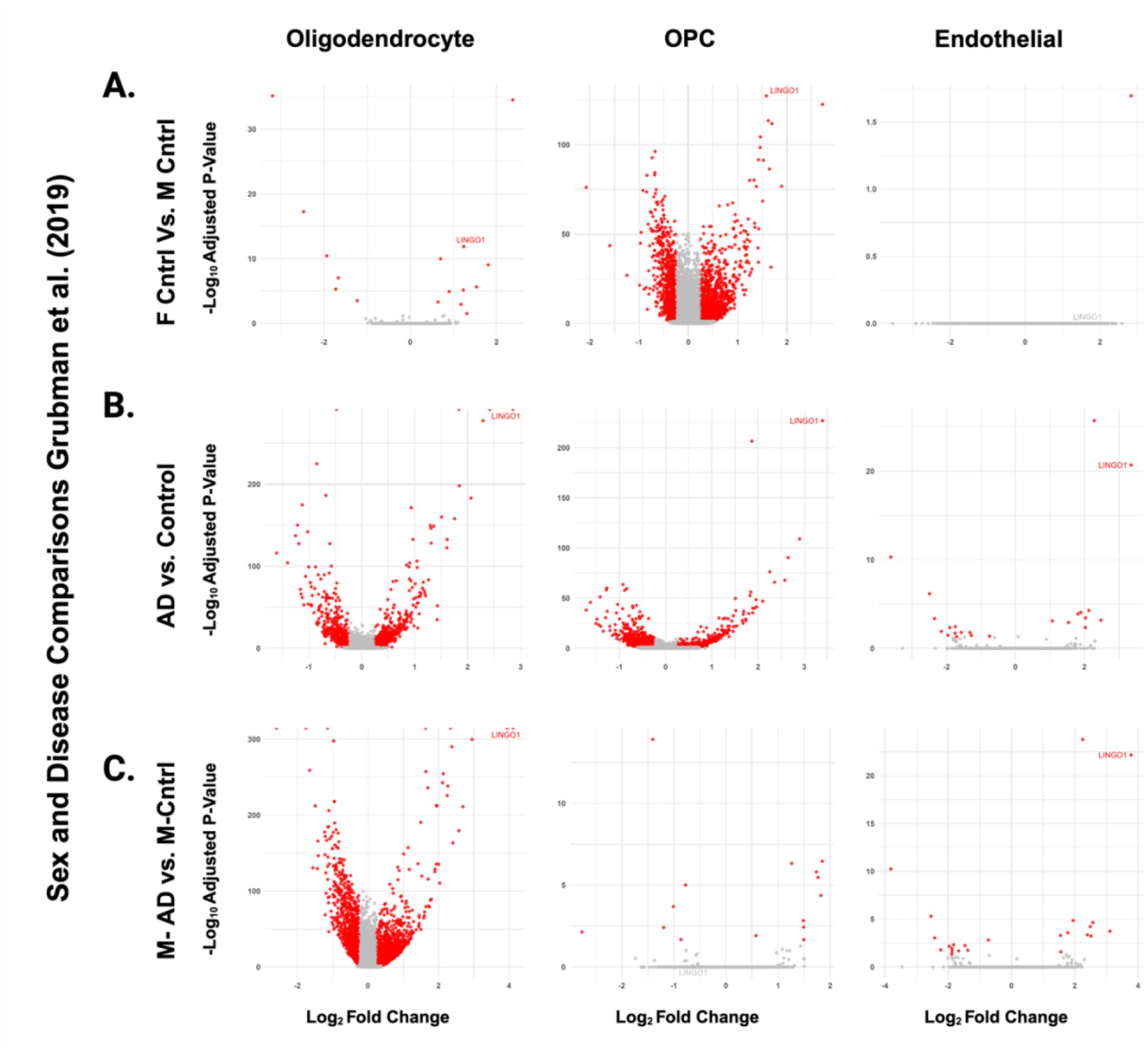

***Figure S4**. Grubman et al. (2019) data summary comparing DEGs from cells in the entorhinal cortex across sex, disease state, and AD diagnosis; (A) Female Control (n=1) v. Male Control patients (n=2), (B) AD patients (n=3) vs. Control patients (n=3) (C) Male AD (n=2) v. Male Control (n=2). DEG gene for these comparisons is shown in oligodendrocytes, OPCs, and endothelial cells. Lingo1 appears as a top significant DEG across nearly all cell types and conditions shown. Genes highlighted in red* are considered significant DEGs *($p<0.05$ and $log_2FC>0.25$).*
